# EMG and Heart Rate Responses Decline within 5 Days of Daily Whole-Body Vibration Training with Squatting

**DOI:** 10.1371/journal.pone.0099060

**Published:** 2014-06-06

**Authors:** André Rosenberger, Anna-Maria Liphardt, Arne Bargmann, Klaus Müller, Luis Beck, Joachim Mester, Jochen Zange

**Affiliations:** 1 Institute of Aerospace Medicine, German Aerospace Center (DLR), Cologne, Germany; 2 Institute of Training Science and Sport Informatics, German Sport University Cologne, Cologne, Germany; 3 Medical Faculty, University of Cologne, Cologne, Germany; University of Zaragoza, Spain

## Abstract

In this study, we examined the acute effects of a 5-day daily whole-body vibration (WBV) training on electromyography (EMG) responses of the m. rectus femoris and m. gastrocnemius lateralis, heart rate (HR, continuously recorded), and blood lactate levels. The purpose of the study was to investigate the adaptation of muscle activity, heart rate and blood lactate levels during 5 days of daily training. Two groups of healthy male subjects performed either squat exercises with vibration at 20 Hz on a side alternating platform (SE+V, n = 20, age  = 31.9±7.5 yrs., height  = 178.8±6.2 cm, body mass  = 79.2±11.4 kg) or squat exercises alone (SE, n = 21, age  = 28.4±7.3 years, height  = 178.9±7.4 cm, body mass  = 77.2±9.7 kg). On training day 1, EMG amplitudes of the m. rectus femoris were significantly higher (P<0.05) during SE+V than during SE. However, this difference was no longer statistically significant on training days 3 and 5. The heart rate (HR) response was significantly higher (P<0.05) during SE+V than during SE on all training days, but showed a constant decline throughout the training days. On training day 1, blood lactate increased significantly more after SE+V than after SE (P<0.05). On the following training days, this difference became much smaller but remained significantly different. The specific physiological responses to WBV were largest on the initial training day and most of them declined during subsequent training days, showing a rapid neuromuscular and cardiovascular adaptation to the vibration stimulus.

## Introduction

Whole-body vibration training (WBVT) has become a popular training method for recreational and athletic training and also in the field of rehabilitation medicine. The acute effects of locally applied vibration and the short- and long-term effects of WBVT have been intensively studied over the last three decades [1]–[6].

When a subject stands on a vibration platform with slightly bent knees, the magnitude of the acceleration (measured by accelerometers) that is transmitted through the body decreases substantially from the vibration platform to the subject's trunk (table 1). It has been suggested that the body sway is mainly reduced at the knee joint by the action of muscles and tendons, which function as a damping system [7]. As a physical consequence of the vibration damping, the leg muscles convert a substantial fraction of the incorporated kinetic energy into heat [8]. In addition leg muscles show an active response to the application of vibration by an increase in the EMG amplitude [9]–[14] and by an increased energy turnover [15]–[17].

**Table 1:** The accelerations at different locations during squat exercises with vibration (SE+V).

Location	Vertical (longitudinal) acceleration (m·s^−2^)	Anterior/Posterior (sagittal) acceleration (m·s^−2^)
Vibration platform	38.1±3.1	-
Ankle	29.6±6.6	14.1±3.9
Patella	3.9±3.5	3.2±4.9
Clavicle	0.5±0.2	0.5±0.2

The acceleration (m·s^−2^) at the vibration platform, the ankle, the patella, and the clavicle, calculated as the root mean squares and averaged over all of the series of squats and all of the training days (means ±SD). Gravity and low-frequency acceleration were removed from the raw signal by a 1-Hz high-pass 3^rd^-order Butterworth filter. The damping of vibration along the body axis in the upward direction includes a decline in the amplitudes and an increasingly equal distribution of the acceleration vector in the anterior/posterior direction.

The combination of mechanical stress and additional muscle activation by WBVT combines commonly known anabolic stimuli. Therefore, WBVT was tested as an alternative or add on to conventional resistive training to counteract muscle atrophy during bed rest. Bed rest serves as a model for unloading and immobility caused by microgravity, disease, or extreme sedentary lifestyle. Daily WBVT at 20 Hz failed to counteract the atrophy of leg muscles during 14 days of bed rest [18]. A combination of WBVT (26 Hz) with high intensity resistive exercise could almost stop muscle loss during 56 days of bed rest [19], [20]. However, specific benefits of resistive training plus WBVT over resistive training alone in terms of muscle volume and function were not found [21]. Thus, more knowledge about the origin and the properties of the augmented muscle activation is necessary to understand the failures of WBVT in counteracting disuse atrophy.

A local vibration stimulus of a single muscle or tendon has been described as leading to a typical tonic vibration reflex (TVR) [22]–[24], which is thought to be predominantly a monosynaptic reflex [25]. During WBVT, a person is standing on a vibrating platform, and thus, vibration is synchronously applied to the mechanoreceptors in all of the leg muscles, tendons, and ligaments as well as the skin. The potential involuntary increase in the muscle activity in response to the vibration component of WBVT likely results from more complex neuronal processes than the classical TVR [26]–[28]. In clinical practice, monosynaptic tendon reflexes are best tested when muscles are fully relaxed. In contrast, the augmented muscle activation by WBVT was recently shown to increase with increasing additional muscle contraction force [6] which underlines that the augmented muscle activation by WBVT is not evoked by a simple reflex.

Typically, the initial body responses to a training stimulus decrease to more moderate levels when training sessions are repeated in a regular manner, e.g., daily. However, the specific adaptation to the vibration component during daily WBVT has not yet been examined. In the present study, we therefore focused on the changes in augmented body responses to daily WBVT added to squat exercises (SE+V) in comparison with squat exercises alone (SE). The muscle activity of the m. rectus femoris and the m. gastrocnemius lateralis (EMG amplitudes and EMG amplitude increases), the autonomic increase of HR and the increase in blood lactate were examined to test for elevated responses during SE+V in comparison with SE. In addition we measured the accelerations from the vibration platform and along the body axis to test the ability of leg muscles to protect the trunk from the vibration stimulus. We hypothesized that the measured parameters would be higher during the 5 consecutive days of daily SE+V in comparison with SE alone.

## Methods

### Experimental Approach to the Problem

The persistence of additional leg muscle activation by WBVT at 20 Hz was an important component of the daily training stimulus applied over longer periods of time during SE+V. This study was designed to validate the persistence of an additional muscle activation by measuring the neuromuscular activity, the autonomic responses, and the anaerobic glycolytic ATP formation to squatting with (SE+V) and without (SE) WBV. Therefore, EMG amplitude, heart rate (HR), and blood lactate were measured in both conditions. During SE+V, the damping of vibration along the body axis was also measured using accelerometers. EMG and blood lactate are used to indicate additional involuntary muscle activation caused by the vibration in addition to the mere work load of SE. The magnitude of the increase in HR reflects the autonomic response to the exercise stimulus and is used here to indicate a potentially increased muscle energy turnover due to the vibration stimulus. EMG was analyzed from the m. rectus femoris which is actively working during squatting and stimulated by vibration via the patella tendon. Furthermore, the EMG was measured from the m. gastrocnemius lateralis which stabilizes the position of the tibia during squatting and is directly stimulated by vibration via the heel and the Achilles tendon. During SE+V, we expected to find predominantly reflex activity in the EMG of the gastrocnemius muscle.

### Subjects

A total of 41 moderately trained healthy male subjects participated in this study (SE+V (n = 20): age  = 31.9±7.5 yrs., height  = 178.8±6.2 cm, body mass  = 79.2±11.4 kg; SE (n = 21): age  = 28.4±7.3 years, height  = 178.9±7.4 cm, body mass  = 77.2±9.7 kg). The mean age, height and body mass values were not significantly different (P>0.05) between the two groups. The study received approval from the Ethics Committee of the North Rhine Medical Association (Ärztekammer Nordrhein), Düsseldorf, Germany. All participants volunteered to participate in this study and gave written informed consent. The subjects were aware that they could withdraw from the study at any time.

The 41 subjects were assigned to the SE+V group or to the SE group in the order of their medical acceptance. Thus, the first 20 subjects were assigned to the group performing SE+V. The subsequent 21 subjects were assigned to the group performing SE. Before onset of the study, subjects had not been familiar with WBVT. The week before the training week, the subjects were familiarized with the experimental setup and the vibration platform, and they performed short squat demonstrations to show that they were able to execute the required movements correctly. Subjects did their individual training at the same time of day for the 5 day training period. The exclusion criteria were as follows: nicotine or drug abuse, chronic disease, regular medication, fractures within the last 6 months and blood donation within the last 2 months. Subjects were asked to drink an appropriate amount of water before the training and to skip exercise/lower leg training for the training week. Also they should not change their normal sleep routine, food intake or drinking habits during the 5 day training period.

### The training protocol

The subjects in both groups trained on 5 consecutive days (Monday to Friday) in our climate-controlled laboratory. On each of the 5 training days, all of the subjects performed one training session of 10×1 min of squats. The speed was controlled at 10 squats/minute by a metronome that was incorporated in the data acquisition system and by the instructions of an operator. The subjects moved downward for 3 s, until a knee angle of approximately 90° was reached, and then upward for 3 s, until a knee flexion angle of approximately 5° was reached. Full extension of the knee was avoided. Between the squat cycles, the subjects rested for 1 min while sitting on a chair. A 5-mm plate was mounted on the vibration platform to raise the subject's heels in order to help subjects to reach the required knee angle at the low squat position without lifting the heel. Furthermore, the subjects were advised to load both legs and the whole sole of each foot equally during the squats. In addition, each subject carried 10% of his individual body weight on a weight belt around his pelvis to increase the muscle tension. The SE+V group trained on a side-alternating vibration platform (Galileo 900, Novotec Medical GmbH, Pforzheim, Germany) at a frequency of 20 Hz and an amplitude of 3–4 mm (6–8 mm peak-to-peak). The SE group performed the same training while the vibration platform was turned off. Both groups performed their interventions barefoot.

### Measurement of Electromyography (EMG) and accelerations by vibration

EMG was recorded from a knee extensor muscle which was involved in the performance of the squats and a plantar flexor muscle which was predominantly stabilizing stance. For EMG records of knee extensors we chose the distal part of the m. rectus femoris, which is affected by the vibration transmitted via the patellar tendon. Furthermore, EMG was recorded from the center of the belly of the m. gastrocnemius lateralis, which is affected by the vibration transmitted via the Achilles tendon. EMG was recorded using bipolar surface electrodes (Type: Blue Sensor – N10A; Ambu GmbH, Bad Nauheim, Germany) on both legs on training days 1, 3, and 5 while the subjects performed the squats. The electrodes were connected by 6-cm cables to a preamplifier (Noraxon USA Inc., Scottsdale, AZ, USA) operating with a frequency band from 5 to 500 Hz. The skin was carefully prepared by shaving and cleaning with alcohol to reduce the resistance between the electrodes to less than 10 kΩ. The distance between the two sensor areas of an electrode pair was 2 cm. To reduce the electromagnetically induced artifacts in the EMG signal, the preamplifiers and the 6-cm cables to the electrodes were tightly fixed to the skin using medical adhesive tape (Leukosilk, BSN medical GmbH, Hamburg, Germany).

On all training days, acceleration (m·s^−2^) of the vibration platform and the resulting forces transmitted along the body axis were recorded using accelerometers (2-axes accelerometer, biovision, Wehrheim, Germany) fixed on bony landmarks with medical glue (medical adhesive spray, Hollister Incorporated, Libertyville, IL, USA) and medical adhesive tape (Leukosilk, BSN medical GmbH, Hamburg, Germany). One accelerometer was fixed on the vibration platform at a position with a vibration amplitude of about 4 mm. This position of the accelerometer was medial from the position where the subjects placed their right halluxes when standing on the vibration platform. Further accelerometers were fixed at the lateral malleolus of the right foot, the center of the patella of the right leg, and on the medial end of the right clavicle. The acceleration was recorded along two axes, the longitudinal body axis, which is vertical when subjects stand upright, and the sagittal axis, which is oriented anterior-to-posterior. Especially during squatting, the accelerometer fixed on the patella would tilt from the vertical towards the anterior/posterior axis by a few degrees. To identify the lower reversal point in the movement as a trigger for the EMG and acceleration analysis at the point of maximum muscle load, the knee angle was monitored using a goniometer (1-axis Goniometer, biovision, Wehrheim, Germany) that was fixed to the lateral side of the knee.

The raw signals from the EMG, the four accelerometers, and the goniometer were recorded with a MyoSystem 1400A (Noraxon USA Inc., Scottsdale, AZ, USA) at a sampling rate of 1000 Hz using the MyoResearch XP software (Version 1.03.05, Noraxon USA Inc., Scottsdale, AZ, USA). The Data Acquisition System Laboratory 7 (DASYLab 7.0, Version 7.00.04, National Instruments Corporation, Austin, TX, USA) with a custom-made program was used to process the raw signals further. From the EMG records of every squat, an interval of 2048 data points before and 2048 data points after the squat minimum was selected (a total of 4096 ms) for a Fast Fourier Transformation. Thus, the portion of the EMG record that was analyzed for every squat contained approximately two-thirds of the EMG signal of a squat cycle but no additional EMG signal from a previous or subsequent squat.

**Figure 1:**
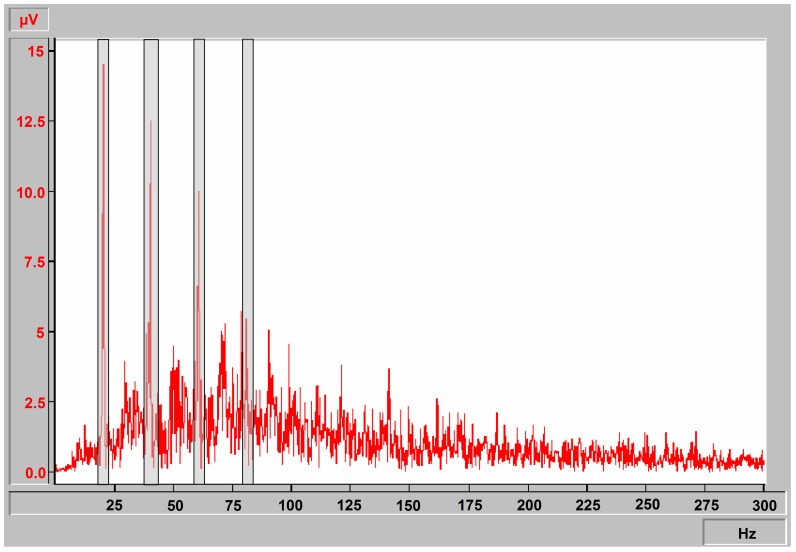
A typical EMG spectrum of a contracting muscle. A typical EMG spectrum of one subject with narrow excluded frequency bands (light gray bars) at 20±2 Hz, 40.5±2.5 Hz, 61±2 Hz, and 82±2 Hz.

In addition, mechano-sensing during WBVT triggers reflex responses of the leg muscles that may become visible as synchronous or non-synchronous EMG activity [9], [23], [24], [26]–[28]. Narrow banded stop filters at the vibration frequency and its harmonics applied to a bipolar surface EMG record erase electrical motion artifacts [27], [29], [30] and H-wave like sum action potentials potentially occurring at the vibration frequency [28]. In this study, we did not apply band stop filters on the time domain of the EMG signal but excluded narrow frequency bands from the EMG spectra. This allows the analysis of the non-synchronous natural EMG activity and the vibration-induced synchronous EMG activity. Therefore, we defined narrow frequency bands of 20.0±2.0 Hz, 40.5±2.5 Hz, 61.0±2.0 Hz, and 82.0±2.0 Hz within the EMG raw spectrum (Figure 1) to discriminate between non-synchronous natural EMG activity and motion artifacts (at the vibration frequency and its harmonics [27]). Because these narrow frequency bands also include natural EMG signal, they were applied to the EMG records of both groups (SE+V and SE). The EMG spectrum outside the narrow frequency bands was analyzed for the sum of amplitudes over the remaining frequencies (µV) and for the median frequencies (Hz). The mean values were calculated for each series of 10 squat cycles. The sum of amplitudes was also calculated for EMG content of the narrow frequency bands to get an estimate of the relative portion of the artifact signal and the natural EMG content by comparing the signals of SE+V and SE.

The same time intervals as used for the EMG signals were selected from the acceleration records for further evaluation. As a result, the acceleration at the vibration platform and the different body parts are presented as the mean values for each series of 10 squat cycles. The vibration platform required 3 seconds to build up to the end frequency of 20 Hz. A 1-Hz high-pass 3^rd^-order Butterworth filter was applied to erase the gravity and low-frequency components from the acceleration raw signal. For each data interval, the magnitude of the acceleration was calculated as the root mean square.

### Measurements of heart rate

A one-channel electrocardiogram (ECG) signal was continuously recorded (BIOPAC Systems Inc., Goleta, CA, USA) at a sampling rate of 2000 Hz before and during the squat exercises on training days 1, 2, 4, and 5. The ECG was analyzed for the HR with custom-made software (Synaptek, Bonn, Germany).

The data from every training session were split into the baseline period (2 minutes at rest directly before the training) and 10 training periods (10×1 minute training). The mean values were determined for each baseline period and training period. Finally, the delta values (the mean of each training period minus the mean of the corresponding baseline period) were calculated. HR (min^−1^) was recorded beat-by-beat and calculated from the R-R intervals in the ECG.

### Blood lactate measurements

The lactate (mmol·l^−1^) levels were determined from blood samples drawn from an antecubital vein (before and directly after the training on every training day). After blood sampling using a fluoride monovette, the blood was centrifuged and blood plasma was stored at 5°C in a refrigerator. Blood lactate was then determined at the latest two days after blood sample collection using an enzymatic colorimetric test kit by ABX Pentra (Rolf Greiner BioChemica GmbH, Flacht, Germany).

### Statistical analyses

Statistical analysis was performed using the software STATISTICA 7 (StatSoft Inc., Tulsa, OK, USA) and SPSS 20 (IBM Corporation, Armonk, NY, USA). All variables were tested for normal distribution. EMG amplitudes were not normally distributed and therefore they were transformed by the logarithm to the base 10. EMG amplitudes and median frequencies, HR, and blood lactate of the SE+V and SE group were compared for effects between interventions, days, and series using two-way repeated measures ANOVA and post-hoc (Tukey's HSD) tests for significance. The slope of the increase in EMG amplitude within the data series from a single training session was tested using a one-way ANOVA. Damping of vibration acceleration along the body axis was tested using two-way repeated measures ANOVA and post-hoc (Tukey's HSD) tests for significance. Differences in subject characteristics were tested using a one-way ANOVA. For all tests, the significance levels were set at P<0.05. In addition, effect sizes using partial eta-squared η^2^ and intraclass correlations were measured. All of the values are presented as the means ± standard deviation (SD). Post-hoc power analysis showed that 20 male subjects in the SE+V group and 21 male subjects in the SE group would provide 82% power to detect the treatment effect with an effect size of 0.4. For the analysis of test-retest-reliability, multi-regression coefficients were calculated for EMG amplitudes of series 1 for all training days in m. rectus femoris (SE+V: r = 0.81; SE: r = 0.74) and in m. gastrocnemius lateralis (SE+V: r = 0.79; SE: r = 0.55). Further multi-regression coefficients were determined for increases in HR between baseline and series 1 (SE+V: r = 0.88; SE: r = 0.89) for all training days and increases in blood lactate after training (SE+V: r = 0.79; SE: r = 0.78) for all training days.

## Results

### Training compliance

All of the subjects in the SE group performed the given exercise as scheduled. On training day 1, two subjects in the SE+V group stopped the training after series 9 and after series 7, because of exhaustion and cramps. The same two subjects plus one additional subject could not complete the training on the first two training days (out of 100 squats per training session, these subjects performed only between 59 and 81 squats on training day 1 and 79 to 89 squats on training day 2); from training day 3 onward, all of the subjects were able to perform the complete training session every day.

### Damping of vibration acceleration

Table 1 summarizes the decline in the mean vertical acceleration during 20 Hz of WBV along the body axis and the transmission of acceleration in the sagittal plane. No significant changes in mean acceleration were found on any of the body segments among the 10 series within a training session or among the 5 training days. Because the subjects stand with their whole feet on the vibration platform, the damping of the longitudinal vibration toward the ankle by the action of the calf muscle and the Achilles tendon was significant but rather small (38.1±3.1 m·s^−2^ at the vibrating platform versus 29.6±6.6 m·s^−2^ at the ankle, P<0.05). A significantly steeper damping compared with the vibration platform was found at the patella (3.9±3.5 m·s^−2^, P<0.05) and the clavicle (0.5±0.2 m·s^−2^, P<0.05), showing a large damping effect within the thigh and the trunk. On all of the body levels, a large portion of the applied vertical (longitudinal) acceleration was redirected into the anterior/posterior (sagittal) direction (Table 1). At the patella and the clavicle, the anterior/posterior acceleration became similar to the vertical component.

### EMG amplitude and median frequency

The EMG amplitudes and the median frequencies of the m. rectus femoris and the m. gastrocnemius lateralis between the left and the right leg were equally included in the statistical analysis.

**Figure 2:**
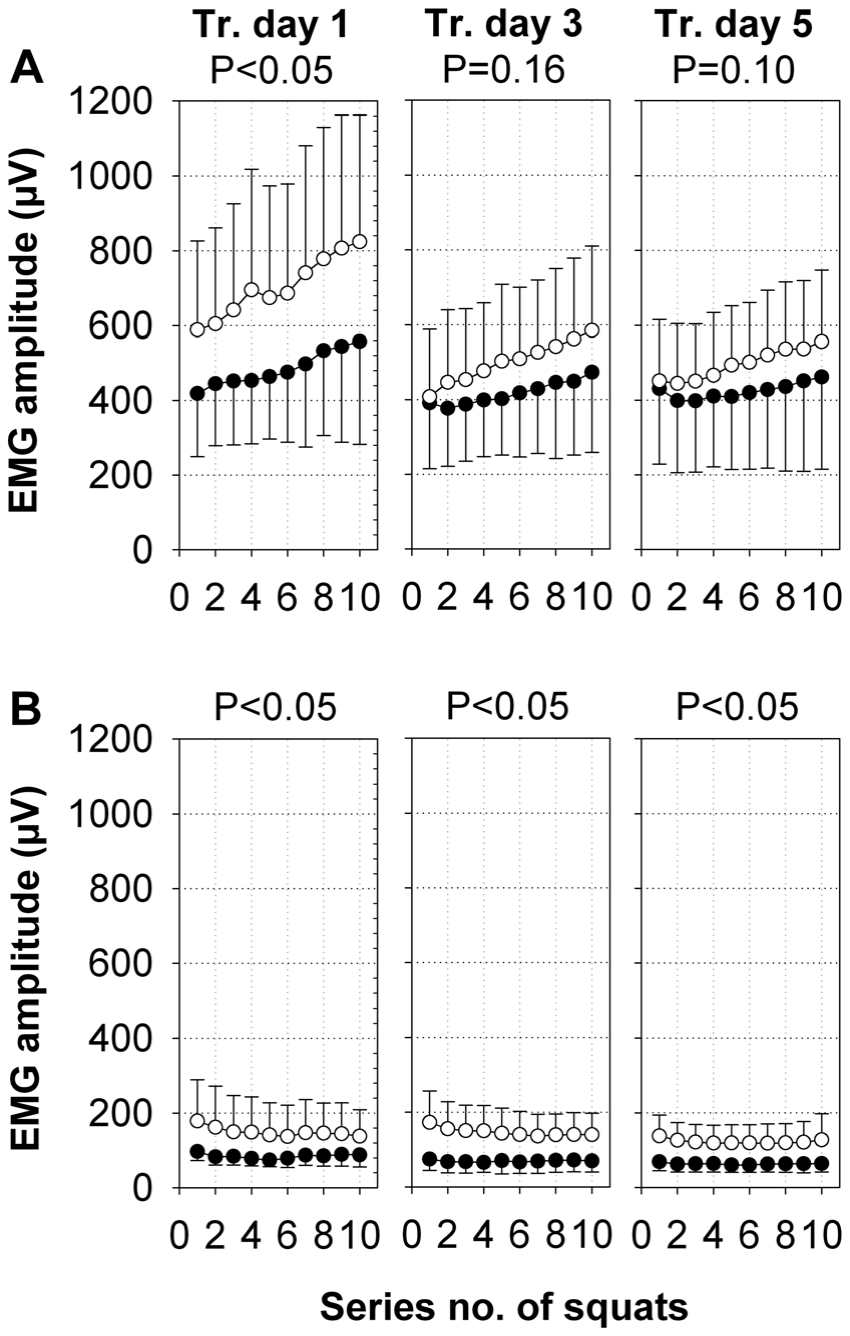
Amplitudes of EMG activity of the m. rectus femoris and m. gastrocnemius lateralis. The amplitudes of the EMG activity (means ±SD, µV) of all of the subjects for the m. rectus femoris (A) and m. gastrocnemius lateralis (B) of both legs during SE+V (○, n = 20) and SE (•, n = 21) on training days 1, 3, and 5. P-values were calculated for intervention group effects by repeated measures ANOVA.

On training day 1, the EMG amplitude of the m. rectus femoris was significantly higher during SE+V than during SE (effect size η^2^ = 0.13, Figure 2A). On training days 3 and 5, the EMG amplitude was not significantly different between SE+V and SE (P = 0.16 with effect size η^2^ = 0.05, P = 0.10 with effect size η^2^ = 0.07, respectively; Figure 2A). The intraclass correlation for all training days was 0.72.

The EMG amplitude of the m. rectus femoris increased during SE+V from series 1 to 10 on all of the training days. The slope of this EMG amplitude started at 7.1±7.1%·series^−1^ on training day 1, dropped to 5.5±4.8%·series^−1^ on training day 3, and finally resulted in 3.5±3.9%·series^−1^ on training day 5. During SE, the slope of the EMG amplitude increase was always significantly lower (training day 1: 3.9±7.0%; training day 3: 2.7±3.4%; training day 5: 1.4±2.5%) than the corresponding value during SE+V.

On all of the training days, the m. gastrocnemius lateralis EMG amplitude of the SE+V group was significantly higher than the corresponding EMG amplitude of the SE group (Figure 2B). The EMG amplitudes of the m. gastrocnemius lateralis showed a significant decrease in both groups by less than 15% between the first and the last series of a training session. The effect sizes were η^2^ = 0.25 on training day 1, η^2^ = 0.51 on training day 3, and η^2^ = 0.50 on training day 5. The intraclass correlation for all training days was 0.75.

Overall, the EMG amplitude during the squats under both conditions in the m. rectus femoris was always 3–4 times higher compared with the EMG amplitude measured in the m. gastrocnemius lateralis on the corresponding training days (Figure 2).

**Figure 3:**
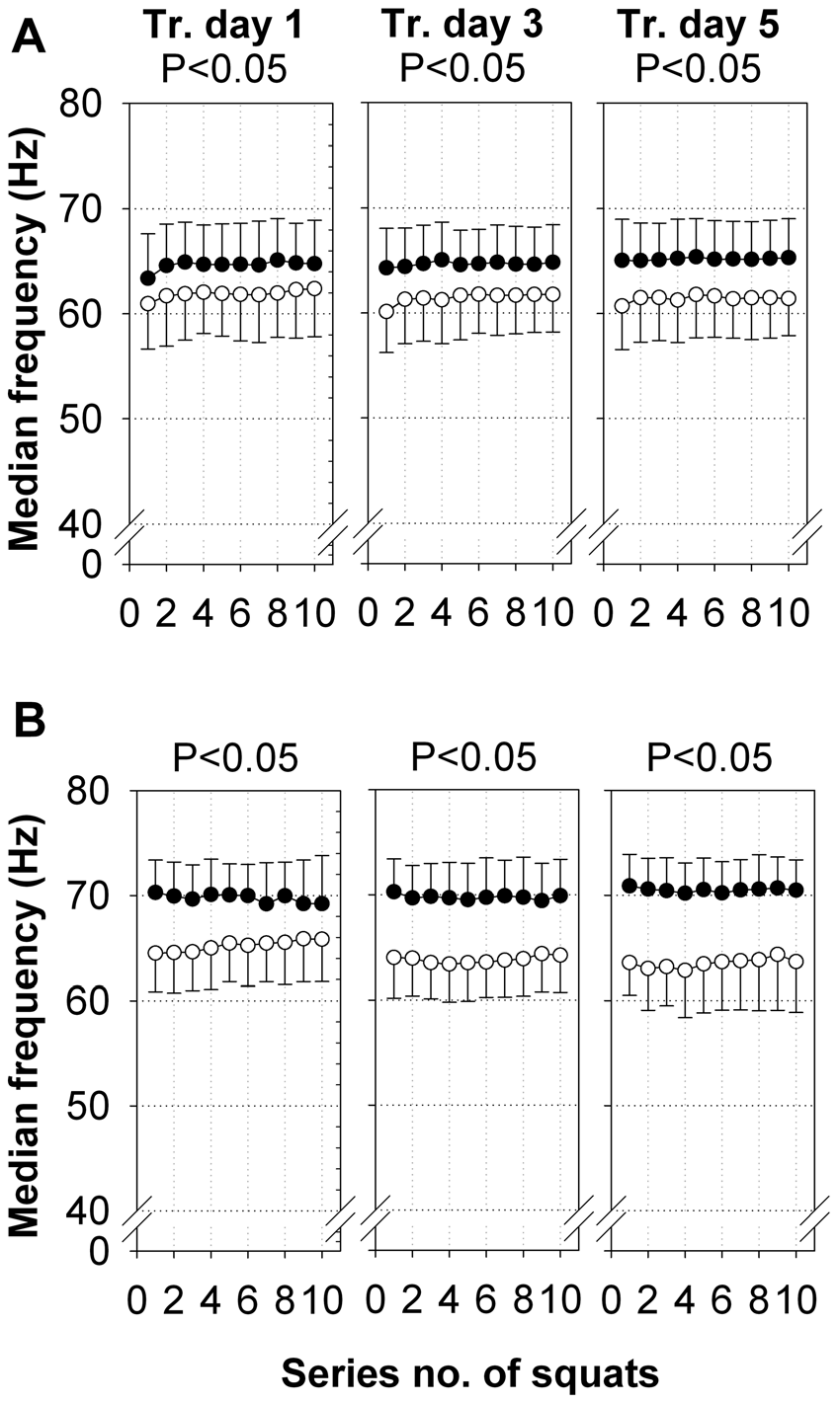
Median frequencies of EMG activity of the m. rectus femoris and m. gastrocnemius lateralis. The median frequencies of the EMG activity (means ±SD, Hz) of all of the subjects for the m. rectus femoris (A) and m. gastrocnemius lateralis (B) of both legs during SE+V (○, n = 20) and SE (•, n = 21) on training days 1, 3, and 5. P-values were calculated for intervention group effects by repeated measures ANOVA.

In both examined muscles, the absolute median frequency of the EMG was significantly lower during SE+V than during SE (Figure 3). This difference between the groups persisted for all 5 training days, and the difference was greater in the m. gastrocnemius lateralis than in the m. rectus femoris (P<0.05). The median frequencies did not change significantly within a training session from series 1 to 10 in either muscle under either condition.

**Figure 4:**
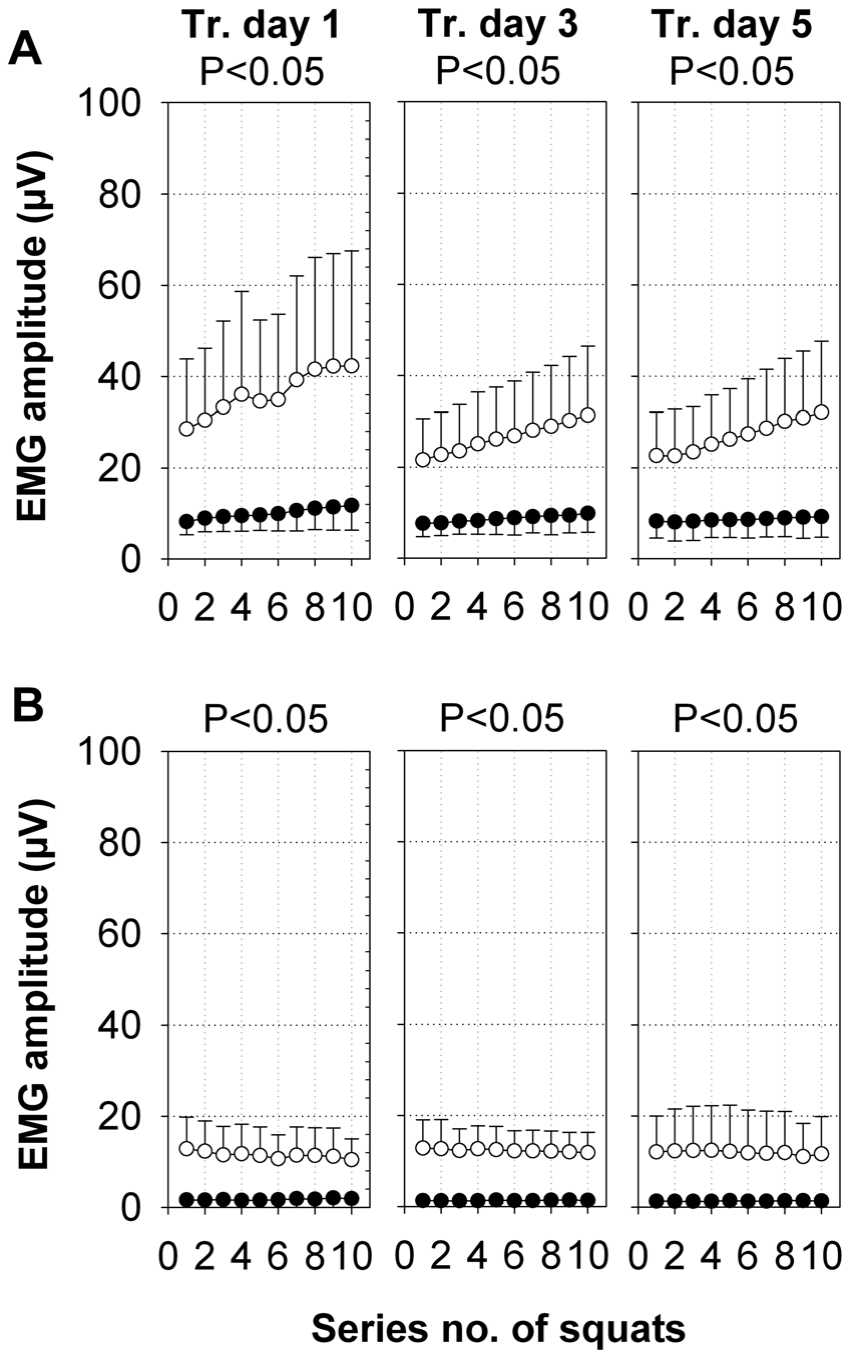
Excluded narrow frequency bands of the m. rectus femoris and m. gastrocnemius lateralis. The amplitudes of the EMG activity within the excluded narrow frequency bands at 20±2 Hz, 40.5±2.5 Hz, 61±2 Hz, and 82±2 Hz (means ±SD, µV) of all of the subjects for the m. rectus femoris (A) and m. gastrocnemius lateralis (B) of both legs during SE+V (○, n = 20) and SE (•, n = 21) on training days 1, 3, and 5. P-values were calculated for intervention group effects by repeated measures ANOVA.

In the SE+V group, the EMG amplitude of the m. rectus femoris in the excluded narrow frequency bands (Figure 4A) was approximately 5% of the intensity of the EMG amplitude of the remaining natural EMG signal (Figure 2A). This EMG amplitude also increased linearly from series 1 to series 10, similar to the analyzed EMG amplitude around the excluded narrow frequency bands. In the SE group, the EMG amplitude of the m. rectus femoris in the excluded narrow frequency bands was approximately 2.5% of the intensity of the EMG amplitude of the remaining natural EMG signal, and it increased slightly from series 1 to series 10. The significantly higher EMG amplitude in the excluded narrow frequency bands of the SE+V group includes both, the non-synchronous natural EMG and the vibration-induced synchronous EMG.

In the SE+V group, the EMG amplitude of the m. gastrocnemius lateralis in the excluded narrow frequency bands (Figure 4B) was approximately 10% of the intensity of the EMG amplitude of the remaining natural EMG signal (Figure 2B). In the SE group, the EMG amplitude of the m. gastrocnemius lateralis in the excluded narrow frequency bands was almost not present. The significantly higher EMG amplitude in the excluded narrow frequency bands of the SE+V group includes both, the non-synchronous natural EMG and the vibration-induced synchronous EMG.

### Heart rate

Because of technical problems with the recording of the HR data (beats per minute, min^−1^), only 19 subjects in the SE+V group and 20 subjects in the SE group could be included in the analysis. There was no difference in baseline HR between the two groups (SE+V: 77±10, SE: 75±7; P>0.05).

**Figure 5:**
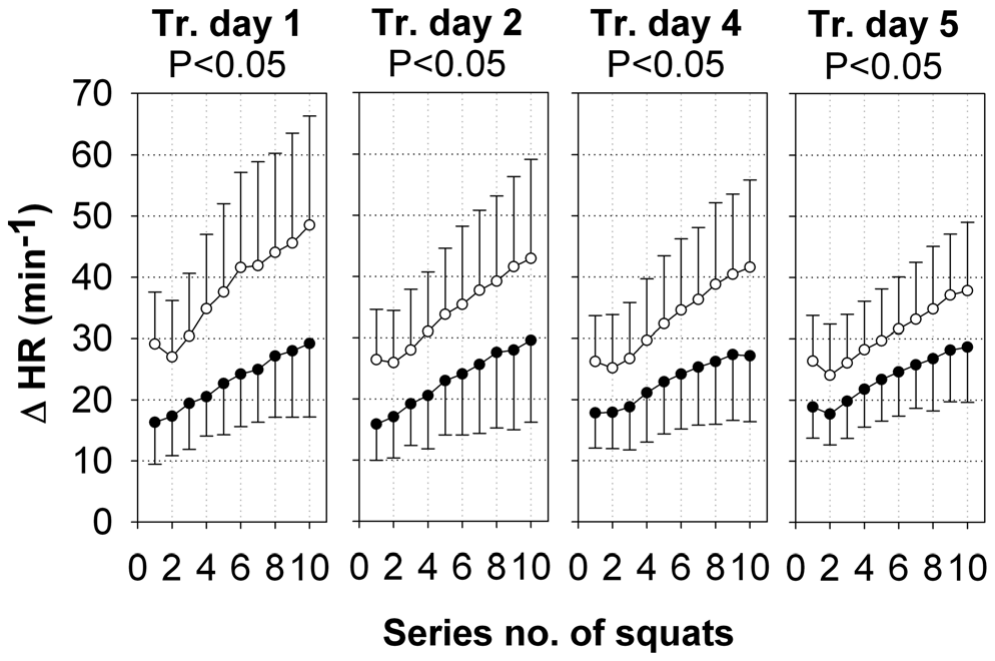
Heart rate changes during squat exercises. The changes from the baseline values (means ±SD) of all of the subjects in the heart rate (HR, min^−1^) during squat exercises in SE+V (○, n = 19) and SE (•, n = 20) on training days 1, 2, 4, and 5. P-values were calculated for intervention group effects by repeated measures ANOVA.

In the SE group, squatting alone caused significant changes in HR compared with the baseline values (Figure 5). On training day 1, HR increased by 16±7 min^−1^ during series 1 and increased further to reach a delta value of 29±12 min^−1^ during series 10 (Figure 5). On the following training days, the HR response to SE was similar during each training session. Between the training days, HR response to training was not significantly different in the SE group.

In the SE+V group, the exercise effects on HR were significantly higher than the corresponding effects in the SE group for all training days (Figure 5). On all of the training days, the HR response during SE+V increased almost linearly from series 1 to series 10, but the slope became less steep from day to day shown by a decreasing delta HR at series 10 (training day 1: 49±18 min^−1^ with effect size η^2^ = 0.33, training day 2: 44±17 min^−1^ with effect size η^2^ = 0.24, training day 4: 41±14 min^−1^ with effect size η^2^ = 0.23, training day 5: 38±12 min^−1^ with effect size η^2^ = 0.20). The intraclass correlation for all training days was 0.78.

### Blood lactate

**Figure 6:**
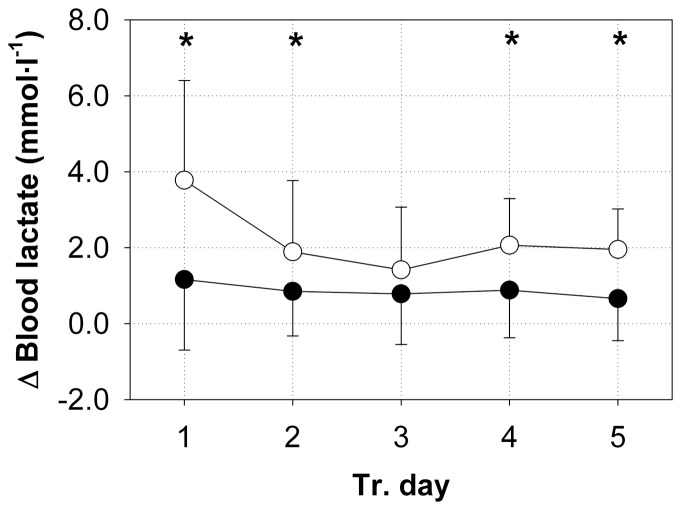
Blood lactate changes during squat exercises. The increase in blood lactate levels (means ±SD, mmol·l^−1^) of all of the subjects during squat exercises in SE+V (○, n = 20) and SE (•, n = 21) on training days 1, 2, 3, 4, and 5. The increase in blood lactate was calculated by the post-training values minus the pre-training values. *Significant difference between SE+V and SE (P<0.05, Tukey's HSD test after ANOVA).

The net blood lactate was calculated as the post-exercise values minus the pre-exercise values. On each training day, the SE group resulted in a net blood lactate level of approximately 1 mmol·l^−1^ (Figure 6). On training day 1, the SE+V group caused a net increase in the blood lactate level of 3.8±2.6 mmol·l^−1^. This augmented blood lactate level in the SE+V group dropped to a net increase of 1.9±1.9 mmol·l^−1^ on training day 2, and it remained almost constant at that level on the following training days. On training days 1, 2, 4, and 5, the net blood lactate levels in the SE+V group were significantly higher than in the SE group.

## Discussion

The major finding of this study was a very early adaptation of EMG activity in the thigh to 20 Hz WBVT. Augmented EMG amplitude in SE+V could be shown during the first training day only, whereas on the third and fifth training day, the EMG amplitude declined to insignificant levels. The weakened potential of daily WBV to increase muscle activity during squatting was confirmed by declining autonomic responses in terms of HR and declining metabolic responses in terms of blood lactate levels. However, in contrast to the vibration effect on EMG, which became insignificant from training day 3 onwards, values of HR and lactate remained significantly higher during vibration on all training days.

The m. gastrocnemius lateralis showed the expected low EMG activity during squatting. Since the gastrocnemius muscle was only minimally loaded by squatting, the augmented amplitudes caused by the vibration stimulus were smaller in the m. gastrocnemius lateralis than in the m. rectus femoris. This supports the finding that the additional activation of a muscle by WBVT increases with increased additional force by a voluntary contraction [6].

In the following we discuss the problems of EMG recording during vibration training, the potential neurophysiological origin of the additional muscle activation by vibration and potential reasons for the rapid adaptation within only a few days of daily training, and finally the potential consequences of this rapid adaptation of the neuromuscular system for the application of vibration as an additional stimulus in conjunction with resistance training using moderate levels of loading.

During WBVT, the recording of EMGs to measure muscle activity is technically challenging. Large artifacts in the EMG signal at the vibration frequency and its harmonics are likely caused by the motion between the skin and the muscle. A bipolar high-pass filtered EMG records such electrical field inhomogeneity when the pair of electrodes attached to the skin is moved (e.g. during WBVT at 20 Hz). These artifacts must be removed from the EMG signal [27], [29]. During WBVT, artifacts with similar amplitudes can be observed in bipolar EMG records from the bellies of the lower and upper leg muscles and from the patella [29]. Therefore, the muscle activity was estimated using the EMG amplitude of the surface EMG, which was split into two portions: the smaller part of the EMG amplitude that was found in the excluded narrow frequency bands corresponding to the vibration frequency and its harmonics, and the larger part that did not correspond to the vibration frequency. In principal, the EMG in the excluded narrow frequency bands contained electrical artifacts and some natural muscle activity. For a comparison, which EMG is synchronized or non-synchronized by the vibration, Ritzmann et al. [28] concluded that the phase relationship between the vibration frequency of the vibration platform and the electrical responses of the leg muscles may support the interpretation that stretch (Hoffmann) reflexes caused by the vibration of the muscle spindles are a major source of vibration-related elements in the EMGs of the leg muscles during WBVT, which indicates a frequency-related active response of the muscles. However, the action potentials of single motor units do not synchronize with each other by the application of a vibration [31]. In conclusion, we suggest that the excluded narrow frequency bands that correspond to the vibration frequency and its harmonics should be considered to be predominantly artifactual.

In the m. rectus femoris, the development of the EMG amplitudes during squatting alone (SE) indicates a moderate adaptation to this type of exercise between training days 1and 5, whereas with WBVT (SE+V), the adaptation was much greater (Figure 2). More specifically, SE+V caused higher EMG amplitudes and faster increases in the EMG amplitude during all of the training days compared with SE. This result indicates that the squat exercises in combination with the vibration stimulus required more conscious attention for the execution of the squats (e.g. due to postural instability and lack of familiarity), which may have led to more poorly coordinated and less-efficient squats. Therefore, the amplitude increase during a training session likely reflects an increase in motor unit recruitment to sustain a constant work load and is commonly observed as an indicator of the development of muscle fatigue. However, a corresponding decrease in the EMG median frequency, which is also reported to be a sign of fatigue [32], was not found in our data. On training days 3 and 5 in SE+V, the EMG amplitude during series 1 had already decreased to the EMG amplitude levels of SE. These decreased EMG amplitudes in SE+V showed a fast adaptation to the additional vibration stimulus on the neuromuscular level already on training day 3. In detail, looking at the percentage increase in the EMG amplitude from series 1 to series 10 in SE+V and SE on training day 1, this increase was reduced to half the size of the respective group on training day 5. These reduced increases in the EMG amplitude after 5 days of training indicate optimization processes (e.g., better inter- and intramuscular coordination to reduce motor unit recruitment and the development of muscle fatigue) in both groups, which are well known to occur in the early phase of an unfamiliar regular training regimen.

The EMG amplitudes of the m. gastrocnemius lateralis did decrease in either training group within their series of squats (Figure 2B). This decrease indicates similar optimization processes in the m. gastrocnemius lateralis as found in the m. rectus femoris. However, the low EMG amplitudes also confirmed that the subjects performed the squats with their feet (and therefore also their heels) flat on the vibration platform such that the m. gastrocnemius lateralis was not weight-bearing but was predominantly used for balance. Therefore, the calf muscle loading was reduced to a minimum during all of the squat cycles. In SE+V, however, the EMG amplitude was approximately twice as high as in SE during all of the training days. The small and persisting effect of vibration on the EMG amplitude of the m. gastrocnemius lateralis may result from 1) an additional increase in muscle contraction from an involuntary unloading of the heel that the subjects were unable to avoid throughout the whole vibration period, although they were advised to load the foot sole equally during the squats and/or 2) electrical artifacts that were not eliminated by filtering, thus, a minor portion of the augmented EMG amplitude may still result from electrical interference by motion artifacts, which likely affected the low amplitudes of EMG from the m. gastrocnemius lateralis during SE+V relatively more than the larger EMG amplitudes of the m. rectus femoris. Therefore, we cannot determine to what extent the increase in the EMG amplitude due to vibration was caused by calf muscle contraction as opposed to interspersed artifacts. Interestingly, the absolute EMG amplitude of the m. gastrocnemius lateralis during SE+V was less than a third of the EMG amplitude of the m. rectus femoris during SE+V on training day 1, which is unlikely to result from a mono-synaptic reflex response, where we would have expected a higher EMG amplitude because the m. gastrocnemius lateralis was closer to the vibration platform and exposed to higher acceleration amplitudes by the vibration. The low EMG amplitude at the m. gastrocnemius lateralis, therefore, hints at the possibility that involuntary muscle responses during WBV could be the complex result of different reflexes initiated by a variety of mechanoreceptors in the leg.

The damping of vibration during WBVT is important for reducing the transmission of kinetic energy to the trunk and for avoiding harmful resonance frequencies for the inner organs, the eyes and the head [7], [30], [33]. Our data show that the magnitude of the vibration applied to our subjects was successfully damped from the ankle to the clavicle. This finding is in line with Pollock et al. [30], who recently showed that at frequencies greater than 15 Hz, the acceleration during WBV decreased from the knee to a minimum at the head. Specifically, our data show that the damping of the vibration was the strongest from the ankle to the patella, where a reduction of the vibration of approximately 87% took place. In the end, an acceleration of only approximately 0.5 m·s^−2^ ( = 0.05 of Earth's gravity g) was measured at the clavicle. However, the acceleration was consistent during all of the series on all of the training days and at all of the measuring points. Therefore, we found an almost constant damping during SE+V. In conclusion, all of the effects of daily SE+V on the EMG amplitudes of the observed knee extensor and plantar flexor muscles did not significantly affect the transmission and damping of vibration along the body axis.

Reflex contractions are well known to occur in muscles stimulated by vibration [22], [25], [28]. Our results on the m. rectus femoris in response to SE+V imply that a major increase in EMG amplitude only occurs on the initial day of training. In continuous daily SE+V, the transmission of the acceleration through the body axis resulting from the vibration was not different between training days. Therefore, it appears that the motor control system learned to use the m. rectus femoris as a more passively operating spring and damping system. The permanent effect of SE+V on the EMG amplitude of the m. gastrocnemius lateralis, if it really showed a physiological response to vibration, may hint at a more constant motor response, likely to avoid resonance frequencies [10]. Desmedt [25] indicated, in his review, the complexity of the vibration reflex due to processes resulting in the modulation of muscle spindles, e.g., by the activity of gamma-motor neurons or the gating process modulating the H-reflex. In addition to the muscle spindles in the muscles, further mechanoreceptors are located in the tendons, the ligaments, and the skin. Thus, very complex neuromuscular responses to the WBV stimulus must be expected. It is also well known that reflex responses to unexpected or new mechanical stimuli are generally stronger than those to familiar stimuli. Only the significantly lower EMG median frequency in SE+V compared with SE in the rectus femoris and the gastrocnemius muscle might indicate an increased synchronization of motor unit firing [34], which would support the idea that the elevation of the EMG amplitude during SE+V may, at least partially, result from the classical TVR.

In our study, blood levels of lactate increased significantly more during SE+V than during SE. This finding indicates an augmented ATP formation by anaerobic glycolysis caused by the vibration stimulus in addition to SE. On training day 1, an absolute blood lactate concentration of 5.4±2.7 mmol·l^−1^ was found in the SE+V group after exercise, which is above the commonly defined anaerobic threshold of 4 mmol·l^−1^. Rittweger et al. [12] found similar lactate levels in their study, in which squats until exhaustion superimposed with 26-Hz WBV and an additional weight of 40% body weight caused absolute lactate values of 5.5±2.7 mmol·l^−1^. Therefore, we suggest that the anaerobic ATP formation and the accompanied overall demand of the muscles to maintain their work during squatting, is assessed similarly for WBVT until exhaustion with an additional weight of 40% of the body weight, as investigated by Rittweger et al. [12], and the sub-maximum WBVT of 20 minutes with an additional weight of 10% of the body weight in our study.

In addition to the response in terms of increased EMG amplitude and blood lactate levels, the autonomic reactions of the cardiovascular system also showed significant higher responses to the WBV stimulus during the training sessions over and above the responses to squats only. On all training days of our study, a significant difference in the HR increase between the training groups was observed, showing a persistent vibration effect on the cardiovascular system. However, the overall increase in HR during each training session declined from training day 1 to training day 5, also indicating a cardiovascular training effect in SE+V. Therefore, we suggest that our finding of significantly higher but constantly declining HR increases in the SE+V group may be the result of an inefficiently adapted autonomic response, which is known to be typical for initial responses to an unfamiliar stimulus.

Other acute physiological reactions may occur due to responses to the unfamiliar vibration stimulus, e.g., by inefficient intermuscular coordination. Rittweger et al. [16] showed that 3 minutes of WBVT with squats increased the oxygen uptake significantly in comparison to squats without vibration. These authors suggested that the increased oxygen consumption was likely to be caused by the elicitation of muscle activity through vibration. In addition, Kerschan-Schindl et al. [35] indicated that the muscular blood circulation in the calf and thigh muscles significantly increased after a 9-minute standing test with 26-Hz WBV. Furthermore, Zange et al. [17] investigated the energy metabolism of the calf muscles during 20-Hz vibrations applied by a pedal during 3 minutes of isometric plantar flexion at 40% of the maximum voluntary contraction. They showed that the vibration stimulus leads to a small increase in phosphocreatine consumption and pH reduction compared with the same isometric contraction without vibration. These studies support our findings of acute increased responses due to the vibration stimulus. However, in our study it appears that the responses decline after the first day of daily WBVT.

In conclusion, within 5 days of daily squat exercises in combination with WBVT, our subjects showed a rapid neuromuscular and hemodynamic adaptation to the whole-body vibration stimulus. On training day 3, the EMG amplitude in the SE+V group was no longer significantly different from the SE group values. In addition, the HR increases during a training session showed a constant decline in the SE+V group throughout all training days. These findings are supported by the net lactate production during SE+V, which was the highest on the first training day and stayed on much lower levels throughout the remaining training days. Despite the intense sensations of the vibration and the rapid passive warm up of the leg muscles [8], this kind of training has only little anabolic potential when performed for several days with a constant vibration frequency and a loading that matches body weight plus 10%. Further studies should test whether e.g. (daily) changes of the vibration frequency and/or additional muscle loading are able to maintain the augmented muscle activation by WBVT and the corresponding potential anabolic effect.
